# Supplementary material for: EEG Correlates of Cognitive Dynamics in Task Resumption After Interruptions: The Impact of Available Time and Flexibility
Source: Eur J Neurosci. 2025 Feb 24;61(4):e70027. doi: 10.1111/ejn.70027 (PMC11851001; doi:10.1111/ejn.70027)
Supplement: Supplementary file 1 — Table S1. Main model using the condition labels as the predictor variables and the angular error as the outcome variable (R 2 = 0.12, adjusted R 2 = 0.09, F = 4.33, p < 0.01). Table S2. Model using the condition labels as predictor variables and the oscillatory beta power prior to the retro‐cue as the outcome variable (R 2 = 0.17, adjusted R 2 = 0.14, F = 6.34, p < 0.001). Table S3. Model that includes the oscillatory beta power as one of the predictor variables and the angular error as the outcome variable (R 2 = 0.34, adjusted R 2 = 0.31, F = 12.15, p < 0.001). Bootstrap CI for interruption effects: [1.45–5.41], for duration effects: [−2.19–1.43], for the interaction: [−5.08–0.3]. Table S4. Model using the condition labels as predictor variables and the oscillatory theta power prior to the retro‐cue as the outcome variable (R 2 = 0.13, adjusted R 2 = 0.10, F = 4.68, p < 0.01). Table S5. Model that includes the oscillatory theta power as one of the predictor variables and the angular error as the outcome variable (R 2 = 0.16, adjusted R 2 = 0.12, F = 4.49, p < 0.01). Bootstrap CI for the interruption effects: [0.01–2.79], for the duration effects: [−0.57–1.34], for the interaction: [−3.88 to −0.01]. Table S6. Model using the condition labels as predictor variables and the oscillatory alpha power prior to the retro‐cue as the outcome variable (R 2 = 0.16, adjusted R 2 = 0.13, F = 5.95, p < 0.001). Table S7. Model that includes the oscillatory alpha power as one of the predictor variables and the angular error as the outcome variable (R 2 = 0.16, adjusted R 2 = 0.12, F = 4.36, p < 0.01). Bootstrap CI for the interruption effects: [−1.59–0.25], for the duration effects: [−1.49–0.31], for the interaction: [0.15–4.4]. Table S8. Main model using the condition (fixed vs. flexible) labels as the predictor variables and the angular error as the outcome variable (R 2 = 0.10, adjusted R 2 = 0.08, F = 4.25, p = 0.02). Table S9. Model using the condition labels as predictor var [file EJN-61-0-s001.docx]

## Supplementary

### Mediation Analysis

In this supplementary section, we present the results of a mediation analysis conducted on various EEG parameters (see Fig. 5 & 6) observed during the analysis. This approach aims to explore the potential mediating effects of specific neural markers (e.g., the oscillatory beta power prior to the retro-cue) in the relationship between experimental conditions and behavioral outcomes. The detailed results of model fits and the bootstrapped confidence intervals of the coefficients are provided to emphasize the underlying mechanisms driving the observed effects in the main study.

#### Model: angular error ~ Interruption + Duration + Interruption:Duration (fixed duration trials)

|  | **ß** | **SE** | **t** | **p** | **95%CI** |
| --- | --- | --- | --- | --- | --- |
| **Intercept** | 14.95 | 1.33 | 11.25 | < .001 | 12.31 – 17.59 |
| **Interruption** | 5.36 | 1.88 | 2.85 | < .01 | 1.63 – 9.09 |
| **Duration** | 0.63 | 1.88 | 0.33 | .74 | -3.11 – 4.36 |
| **Interruption*Duration** | -1.22 | 2.66 | -0.46 | .65 | -6.50 – 4.05 |

***Table 1.*** Main model using the condition labels as the predictor variables and **the angular error** as the outcome variable (R^2^ = 0.12, adjusted R^2^ = 0.09, *F* = 4.33, *p* < .01).

#### Outcome: Oscillatory Beta Power

|  | **ß** | **SE** | **t** | **p** | **95%CI** |
| --- | --- | --- | --- | --- | --- |
| **Intercept** | -0.95 | 0.17 | -5.58 | < .001 | -1.29 – -0.62 |
| **Interruption** | 0.86 | 0.24 | 3.56 | .001 | 0.38 – 1.34 |
| **Duration** | -0.09 | 0.24 | -0.36 | .72 | -0.57 – 0.39 |
| **Interruption*Duration** | -0.60 | 0.34 | -1.76 | .08 | -1.27 – 0.08 |

***Table 2.*** Model using the condition labels as predictor variables and **the oscillatory beta power** prior to the retro-cue as the outcome variable (R^2^ = 0.17, adjusted R^2^ = 0.14, *F* = 6.34, *p* < .001).

#### Mediator: Oscillatory Beta Power

|  | **ß** | **SE** | **t** | **p** | **95%CI** |
| --- | --- | --- | --- | --- | --- |
| **Intercept** | 18.65 | 1.33 | 14.0 | < .001 | 16.01 – 21.30 |
| **Interruption** | 2.02 | 1.74 | 1.16 | .25 | -1.44 – 5.48 |
| **Duration** | 0.97 | 1.64 | 0.59 | .56 | -2.29 – 4.22 |
| **Interruption*Duration** | 1.11 | 2.35 | 0.47 | .64 | -3.56– 5.77 |
| **Beta** | 3.90 | 0.69 | 5.61 | < .001 | 2.52 – 5.27 |

***Table 3.*** Model that includes the oscillatory beta power as one of the predictor variables and the angular error as the outcome variable (R^2^ = 0.34, adjusted R^2^ = 0.31, *F* = 12.15, *p* < .001). Bootstrap CI for interruption effects: [1.45 – 5.41], for duration effects: [-2.19 – 1.43], for the interaction: [-5.08 – 0.3].

#### Outcome: Oscillatory Theta Power

|  | **ß** | **SE** | **t** | **p** | **95%CI** |
| --- | --- | --- | --- | --- | --- |
| **Intercept** | -0.20 | 0.14 | -1.50 | .14 | -0.47 – 0.07 |
| **Interruption** | 0.56 | 0.19 | 2.92 | < .01 | 0.18 – 0.94 |
| **Duration** | 0.12 | 0.19 | 0.63 | 0.53 | -0.26 – 0.50 |
| **Interruption*Duration** | -0.81 | 0.27 | -2.96 | < .01 | -1.35 - -0.27 |

***Table 4.*** Model using the condition labels as predictor variables and **the oscillatory theta power** prior to the retro-cue as the outcome variable (R^2^ = 0.13, adjusted R^2^ = 0.10, *F* = 4.68, *p* < .01).

#### Mediator: Oscillatory Theta Power

|  | **ß** | **SE** | **t** | **p** | **95%CI** |
| --- | --- | --- | --- | --- | --- |
| **Intercept** | 15.38 | 1.32 | 11.64 | < .001 | 12.75 – 17.997 |
| **Interruption** | 4.20 | 1.93 | 2.18 | .03 | 0.37 – 8.02 |
| **Duration** | 0.37 | 1.85 | 0.20 | .84 | -3.3 – 4.04 |
| **Interruption*Duration** | 0.43 | 2.72 | 0.16 | .88 | -4.98 – 5.83 |
| **Theta** | 2.08 | 0.98 | 2.12 | .04 | 0.14 – 4.02 |

***Table 5.*** Model that includes the oscillatory theta power as one of the predictor variables and the angular error as the outcome variable (R^2^ = 0.16, adjusted R^2^ = 0.12, *F* = 4.49, *p* < .01). Bootstrap CI for the interruption effects: [0.01 – 2.79], for the duration effects: [-0.57 – 1.34], for the interaction: [-3.88 – -0.01].

#### Outcome: Oscillatory Alpha Power

|  | **ß** | **SE** | **t** | **p** | **95%CI** |
| --- | --- | --- | --- | --- | --- |
| **Intercept** | 0.48 | 0.16 | 2.92 | < .01 | 0.15 – 0.79 |
| **Interruption** | -0.25 | 0.23 | -1.07 | .29 | -0.70 – 0.21 |
| **Duration** | -0.26 | 0.23 | -1.14 | 0.26 | -0.72 – 0.19 |
| **Interruption*Duration** | 1.09 | 0.32 | 3.37 | .001 | 0.45 – 1.74 |

***Table 6.*** Model using the condition labels as predictor variables and **the oscillatory alpha power** prior to the retro-cue as the outcome variable (R^2^ = 0.16, adjusted R^2^ = 0.13, *F* = 5.95, *p* < .001).

#### Mediator: Oscillatory Alpha Power

|  | **ß** | **SE** | **t** | **p** | **95%CI** |
| --- | --- | --- | --- | --- | --- |
| **Intercept** | 14.17 | 1.37 | 10.38 | < .001 | 11.46 – 16.88 |
| **Interruption** | 5.77 | 1.86 | 3.1 | .003 | 2.08 – 9.47 |
| **Duration** | 1.06 | 1.86 | 0.57 | .57 | -2.64 – 4.76 |
| **Interruption*Duration** | -3.03 | 2.77 | -1.10 | .28 | -8.53 – 2.46 |
| **Alpha** | 1.66 | 0.82 | 2.01 | .05 | 0.02 – 3.295 |

***Table 7.*** Model that includes the oscillatory alpha power as one of the predictor variables and the angular error as the outcome variable (R^2^ = 0.16, adjusted R^2^ = 0.12, *F* = 4.36, *p* < .01). Bootstrap CI for the interruption effects: [-1.59 – 0.25], for the duration effects: [-1.49 – 0.31], for the interaction: [0.15 – 4.4].

#### Model: angular error ~ Interruption + Flexibility + Interruption:Flexibility (fixed trials averaged)

|  | **ß** | **SE** | **t** | **p** | **95%CI** |
| --- | --- | --- | --- | --- | --- |
| **Intercept** | 15.26 | 1.39 | 10.99 | < .001 | 12.5 – 18.03 |
| **Interruption** | 4.75 | 1.96 | 2.42 | .02 | 0.84 – 8.67 |
| **Flexibility** | 0.20 | 0.98 | 0.20 | .84 | -1.76 – 2.15 |
| **Interruption*Flexibility** | 0.20 | 0.98 | 0.20 | .84 | -1.76 – 2.15 |

***Table 8.*** Main model using the condition (fixed vs flexible) labels as the predictor variables and **the angular error** as the outcome variable (R^2^ = 0.10, adjusted R^2^ = 0.08, *F* = 4.25, *p* = .02).

#### Outcome: Oscillatory Alpha/Beta Power

|  | **ß** | **SE** | **t** | **p** | **95%CI** |
| --- | --- | --- | --- | --- | --- |
| **Intercept** | -0.77 | 0.20 | -3.94 | < .001 | -1.16 - -0.38 |
| **Interruption** | 0.38 | 0.28 | 1.36 | .18 | -0.17 – 0.93 |
| **Flexibility** | -0.24 | 0.14 | -1.76 | .08 | -0.52 – 0.03 |
| **Interruption*Flexibility** | -0.24 | 0.14 | -1.76 | .08 | -0.52 – 0.03 |

***Table 9.*** Model using the condition labels as predictor variables and **the oscillatory alpha/beta power** after the retro-cue as the outcome variable (R^2^ = 0.05, adjusted R^2^ = 0.02, *F* = 1.70, *p* = .19).

#### Mediator: Oscillatory Alpha/Beta Power

|  | **ß** | **SE** | **t** | **p** | **95%CI** |
| --- | --- | --- | --- | --- | --- |
| **Intercept** | 17.45 | 1.41 | 12.34 | < .001 | 14.63 – 20-27 |
| **Interruption** | 3.68 | 1.83 | 2.01 | .05 | 0.02 – 7.34 |
| **Flexibility** | 0.89 | 0.93 | 0.96 | .34 | -0.96 – 2.73 |
| **Interruption*Flexibility** | 0.89 | 0.93 | 0.96 | .34 | -0.96 – 2.73 |
| **Alpha/Beta** | 2.84 | 0.77 | 3.67 | < .001 | 1.3 – 4.38 |

***Table 10.*** Model that includes the oscillatory alpha/beta power as one of the predictor variables and the angular error as the outcome variable (R^2^ = 0.25, adjusted R^2^ = 0.22, *F* = 7.81, *p* < .001). Bootstrap CI for the interruption effects: [-0.48 – 2.87], for the flexibility effects: [-1.7 – 0.1], for the interaction: [-1.7 – 0.1].
